# Blunt Traumatic Cervical Vascular Injury Without any Modified Denver Criteria

**DOI:** 10.5811/cpcem.2018.4.37719

**Published:** 2018-06-26

**Authors:** Jed T. Ritter, Chadd K. Kraus

**Affiliations:** Geisinger Health System, Department of Emergency Medicine, Danville, Pennsylvania

## Abstract

Blunt traumatic cervical vascular injury (BCVI) is challenging to recognize, but it is a potentially devastating entity that warrants attention from emergency physicians. Injury to the vertebral or carotid artery can result in a delayed manifestation of neurologic injury that may be preventable if promptly recognized and treated. The modified Denver Criteria are frequently used to guide imaging decisions for BCVI; however, injuries can still be missed. We present a case of BCVI in a trauma patient whose initial presentation evaded standard screening criteria, illustrating the need for a high index of suspicion for BCVI in blunt trauma.

## INTRODUCTION

Blunt traumatic cervical vascular injury (BVCI) is challenging to recognize and is a potentially devastating clinical entity that warrants special attention from emergency physicians. Injury to the vertebral or carotid artery can result in a delayed manifestation of neurologic injury that may be preventable if promptly recognized and treated. The modified Denver Criteria are frequently used to guide imaging decision for BCVI, however, injuries can still be missed even using these criteria. We present a case of BCVI in a trauma patient whose initial presentation evaded standard screening criteria, illustrating the need for a high index of suspicion for BCVI in blunt trauma.

## CASE REPORT

A 26-year-old female presented to a Level I trauma center after a motorcycle crash in which she was the unhelmeted passenger thrown from the vehicle. The patient did lose consciousness and was noted to be briefly confused on scene. Her right shoulder had a palpable deformity and she had difficulty moving the right upper extremity, but she denied other symptoms and was transported to our facility via ground emergency medical services. Upon arrival, the patient was in no distress, alert and oriented, and reported only pain in the right shoulder.

Initial vital signs were temperature of 36.7° Celsius, heart rate 107 beats per minute, blood pressure 102/57 mmHg, respiratory rate 18 breaths per minute, and 100% oxygen saturation on room air. She was evaluated by standard trauma protocol. Computed tomography (CT) imaging of the head, cervical spine, and chest/abdomen/pelvis were significant only for a right anterior shoulder dislocation. The patient was treated symptomatically and preparations were made to perform procedural sedation to reduce the shoulder dislocation. Prior to sedation, the patient developed an abrupt change in mental status. Her right pupil became fixed and dilated. She became aphasic, and her right side became flaccid. The patient was immediately intubated based on Glasgow Coma Scale (GCS) of 7 and rapid deterioration of her clinical status.

A repeat CT head was obtained and revealed a hyperdense left middle cerebral artery (MCA). Neurosurgery and neurology were both immediately consulted. CT angiography (CTA) of the head and neck revealed a left internal carotid dissection with tandem embolus to the proximal left MCA. A tandem occlusion is defined by injury that results in cervical carotid artery dissection, as well as embolic occlusion of a large intracranial artery. This type of vascular occlusion typically does not respond well to thrombolysis.^1^ Given the confirmed presence of a tandem occlusion in our patient, a discussion regarding the utility of thrombolytics was held. Neurosurgery opted to perform endovascular mechanical thrombectomy and stenting of the internal carotid artery. Diagnostic cerebral angiogram revealed complete revascularization of the distal left MCA territory. The patient was subsequently admitted to the intensive care unit. There, her course was uncomplicated, and by discharge on hospital day 18 the patient had regained a significant amount of independent function.

## DISCUSSION

Blunt cervical vascular injury (BCVI) is a term used to include injuries to the carotid and vertebral arteries. Although BCVI is rare, it is an entity that can lead to devastating outcomes, including stroke and death.^2^ Current estimates put the incidence of BCVI between 1–2% of all blunt trauma.^3^ If recognized promptly treatments exist for BCVI. Current treatment strategies for BCVI range from antiplatelet and anticoagulation therapy to endovascular stents and mechanical thrombectomy depending on the clinical scenario.

Screening for BCVI is heterogenous. Imaging modalities (e.g., CTA) have improved, 4 but they are inadequate for screening 5 and should not be relied upon exclusively for diagnosis.^6^ One commonly used screening tool, the modified Denver Criteria, suggests using CTA to evaluate for vascular injury in patients with any of the following: unexplained neurologic deficit; arterial hemorrhage, cervical bruit or thrill, infarct on head CT, basilar skull fracture on imaging, expanding neck hematoma; seatbelt abrasion on the neck; GCS less than or equal to 8 in association with blunt trauma; cervical spine fracture; Le Fort II or III facial fractures; or hanging with anoxic brain injury.^7^,^8^ In the case of our patient, she did not have any notable signs or symptoms to prompt early CTA screening for BCVI as suggested by the Denver Criteria. An abrupt onset of new symptoms prompted the imaging, which revealed a complete occlusion of the left internal carotid artery, as well as a tandem embolus to the proximal left MCA.

Treatment for BCVI is dependent upon the severity of the injury. The Blunt Carotid Arterial Injury Grading Scale is the most commonly used tool to stratify vascular injury. It uses a scale from I to V: I represents luminal irregularity or dissection with less than 25% narrowing; II represents greater than 25% narrowing, intraluminal thrombus, or raised flap; III represents pseudoaneurysm; IV represents occlusion; and V represents transection with active extravasation. Management for lower-grade injuries most often consists of antiplatelet or anticoagulation regimens. Multiple studies have shown no clear benefit between antiplatelet and anticoagulation therapy. Management and optimal treatment depends on the clinical scenario. For example, the Eastern Association for the Surgery of Trauma recommends that patients with grade III or greater vascular injuries be considered for operative intervention as they rarely respond to antithrombotic therapy.^10^

Multiple studies have been conducted comparing traditional vs. angio-interventional therapy, without clear superiority of one modality. In the case of our patient, CTA revealed a grade IV dissection with associated proximal MCA embolism. Recent case studies have shown promising results regarding the use of mechanical thrombectomy in association with proximal stenting of the injured vessel.^11^–^17^ Thrombolytics have a limited role in this situation. Endovascular therapy offers an alternative approach, which in the case of our patient provided complete revascularization and a favorable outcome.

**CPC-EM Capsule**

What do we already know about this clinical entity?

Injury to the vertebral or carotid artery can result in a delayed manifestation of neurologic injury that may be preventable if promptly recognized and treated.

What makes this presentation of disease reportable?

We present a case of Blunt cervical vascular injury (BCVI) in a trauma patient whose initial presentation evaded standard screening criteria, illustrating the need for a high index of suspicion for BCVI in blunt trauma.

What is the major learning point?

The Modified Denver Criteria are frequently used to guide imaging decisions for BCVI, however, injuries can still be missed.

How might this improve emergency medicine practice?

*This case demonstrates the importance of considering BCVI in the setting of trauma, even in the absence of the Denver Criteria for* computed tomography angiography*.*

## CONCLUSION

BCVI has gained significant attention in the recent literature, and yet is frequently not recognized in a timely fashion. This case demonstrates the importance of considering BCVI in the setting of trauma, even in the absence of the Denver Criteria for CTA. Current screening criteria and imaging modalities can still miss injuries, delayed diagnosis and treatment leading to devastating sequelae, including death.

Documented patient informed consent and/or Institutional Review Board approval has been obtained and filed for publication of this case report.
